# SLE and APS

**DOI:** 10.1186/1546-0096-12-S1-I19

**Published:** 2014-09-17

**Authors:** Tadej Avcin

**Affiliations:** 1 Department of Allergology, Rheumatology and Clinical Immunology, University Children's Hospital, University Medical Center Ljubljana, Ljubljana, Slovenia

## 

Childhood-onset systemic lupus erythematosus (cSLE) represents 15-20% of all SLE cases and is in general associated with a more aggressive disease course and more rapid damage accrual than adult-onset SLE. Disease expression varies according to ethnicity, with more severe disease course in non-Caucasian ethnic groups. The majority of patients with cSLE develop damage within 5-10 years of disease onset, most frequently involving the musculoskeletal, ocular, renal and central nervous systems. Premature atherosclerosis and osteoporosis have become increasingly prevalent comorbidities in cSLE patients.

Treatment of cSLE is challenging and is further complicated by an unpredictable disease course, adolescent noncompliance and long requirement for therapy. New therapeutic regimens combining immunosuppressive agents and targeted B-cell depletion often provide improved disease control and follow the oncologic model of remission induction and maintenance therapy. Management of children with SLE must include also prevention of medication side effects on growth, delayed puberty, development and fertility. Optimal management of an adolescent with SLE should take into account also patient’s quality of life, psychosocial development and organization of successful transition from pediatric to adult care.

The antiphospholipid antibody syndrome (APS) is a multisystemic autoimmune disease characterized by thromboembolic events, pregnancy morbidity, hematologic, dermatologic, neurologic and other manifestations in the presence of elevated titers of antiphospholipid antibodies (aPL). APS may occur as an isolated clinical entity (primary APS) or in association with autoimmune diseases, infections and malignancies. Multiple pathogenic mechanisms have been proposed by which aPL may predispose to thrombosis including interaction between aPL and endothelial cells, platelets, monocytes, activation of the complement system, and interaction with the proteins involved in the regulation of the coagulation cascade.

Management in all patients with APS include avoidance of additional risk factors for thrombosis. Patients with persistently positive aPL, in particular those with lupus anticoagulants (LA), have a high risk for recurrent thrombosis and should receive long-term anticoagulation with warfarin. The standard treatment in APS patients with venous or non-cerebral arterial thromboembolism consist of oral anticoagulation at a target INR of 2.0-3.0. However, it is essential to individualize treatment according to the presence of additional thrombophilic risk factors and the aPL profile (multiple aPL antibodies, high titers of aCL and/or anti-β2GPI, presence of LA). An improved understanding of the pathogenic mechanisms by which aPL induce thrombosis has suggested some innovative treatments such as new anticoagulant and antiplatelet drugs, hydroxychloroquine, statins, complement inhibitors, rituximab and other targeted therapies.

## Disclosure of interest

None declared.

